# COVID-19 Vaccine Hesitancy Prevalence and Predictors among the Students of Jazan University, Saudi Arabia Using the Health Belief Model: A Cross-Sectional Study

**DOI:** 10.3390/vaccines10020289

**Published:** 2022-02-14

**Authors:** Husameldin Elsawi Khalafalla, Maria Zenaida Tumambeng, Majidah Hussain Asiri Halawi, Enas Mohammed Ali Masmali, Thekra Badr Mohammed Tashari, Fatimah Hasser Abdullah Arishi, Roaa Hassan Mohammed Shadad, Sarah Zaki Abdullah Alfaraj, Shroog Mohammed Ali Fathi, Mohamed Salih Mahfouz

**Affiliations:** Faculty of Medicine, Jazan University, Jazan 45142, Saudi Arabia; hkhalafalla@jazanu.edu.sa (H.E.K.); mmtumambing@dlshsi.edu.ph (M.Z.T.); 201803589@stu.jazanu.edu.sa (M.H.A.H.); 201803572@stu.jazanu.edu.sa (E.M.A.M.); 201803581@stu.jazanu.edu.sa (T.B.M.T.); 201803573@stu.jazanu.edu.sa (F.H.A.A.); 201803699@stu.jazanu.edu.sa (R.H.M.S.); 201808487@stu.jazanu.edu.sa (S.Z.A.A.); 201803702@stu.jazanu.edu.sa (S.M.A.F.)

**Keywords:** vaccine acceptance, vaccine hesitancy, anti-vaccination, vaccine intention, health belief model, survey, Jazan University, COVID-19, coronavirus, SARS-CoV-2

## Abstract

Vaccination has a major role in the control of the COVID-19 pandemic. The behavior toward accepting the COVID-19 vaccine is complex and multifactorial, while the level of acceptance and hesitancy depends on many factors. This study aims to measure the level of vaccine acceptance among Jazan University students and identify its predictors. In this cross-sectional study, an online questionnaire based on the health belief model (HBM) was sent through social media “WhatsApp” to two strata of students selected by convenient sampling. The overall desire to get vaccinated was noted in 83.6% of participants, and 16.4% reported no desire to be vaccinated. The constructs of the HBM were shown to significantly predict vaccine acceptance with the exception of “perceived susceptibility”. In conclusion, the level of acceptance of the COVID-19 vaccine among Jazan University students was relatively high. The HBM constructs are important predictors of the vaccination behavior with the exception of the construct “perceived susceptibility”.

## 1. Introduction

Coronavirus disease 2019 (COVID-19) was declared a pandemic by the World Health Organization on 11 March 2020, approximately three months after the first reported case in Hubei Province, Wuhan, China [1]. At the end of August 2021, the virus had affected more than 200 million people and claimed more than 4 million lives [2].

Several measures were adopted to slow the spread of COVID-19 such as wearing face masks, physical distancing, limiting social gatherings, and lockdown for small and large territories. There were unprecedented efforts to develop effective vaccines, which are anticipated to have an indispensable role in controlling the disease. The efforts were characterized by a faster pace, which resulted in many institutions completing the steps for applying for approval by relevant authorities in a relatively short period of time compared to other older vaccines, and (relatively) new technologies were applied to vaccine development [3]. A herd immunity of approximately 60–72% is required to block virus transmission [4,5]. The newly developed vaccines have been subject to numerous misconceptions, mistrust in scientific information, and challenges in planning, all of which are to be expected to accompany new discoveries [6]. The rapid spread of information and misinformation in the face of a multiplicity of social media outlets resulted in creation of an “infodemic” [7]. Vaccine hesitancy poses a real threat to efforts to control the pandemic globally [8,9].

According to the definition by the Strategic Advisory Group of Experts on Immunization (SAGE) of the World Health Organization, “Vaccine hesitancy refers to delay in acceptance or refusal of vaccination despite the availability of vaccination services” [10] (p. 4163).

The level of vaccine acceptance has been well documented, even before the approval of the potential vaccines, and was found to vary greatly among different populations. Acceptance rates as high as 90% have been reported in Ecuador [11], China [12], and Indonesia [13], and rates as low as 30% have been reported in Kuwait and Jordan [14]. In the Kingdom of Saudi Arabia, a national survey reported a vaccine acceptance level of 64.7% [15]. Among students, a low level of acceptance (34.9%) was reported in Jordan [16], whereas greater than 80% of students in Italy [17] and China [18] expressed willingness for vaccination.

Many factors that shape behavioral intentions and actions have been cited in the literature. Vaccine safety is a great concern, especially in the face of the unprecedented speed of development and approval. Those who are more likely to get vaccinated are those with higher income, better education, males, older age, employed, and health care workers, and the interesting phenomenon of political views has influenced vaccine acceptance, especially in the United States [19].

In the Kingdom of Saudi Arabia (KSA), COVID-19 affected 638,327 people and claimed 8914 lives as of January 2022 [20]. At the time of data collection, vaccination efforts had started approximately six weeks earlier, and there were no vaccination mandates upon entry of public places, including universities. However, strict physical distancing and mask-wearing mandates had been enacted in many cases. By 31 August 2021, greater than 36 million doses of vaccines were administered, and more than 14,500,000 people received two doses of Astra-Zeneca and/or Pfizer vaccines [21].

Behavior toward accepting the COVID-19 vaccine is complex and multifactorial; thus, the levels of acceptance and hesitancy vary across time and place with some countries of the Eastern Mediterranean (EM) and/or the gulf regions being at the bottom of the list [14]. Given that vaccination is an intervention with good potential and other contributors to bring the pandemic under control, vaccine hesitancy is the most imminent threat to achieving that goal. Exploring levels of vaccine hesitancy and its predictors is thus an urgent research priority [22], especially in the EM region [23]. This study aims to determine the level of vaccine acceptance among students of Jazan University, KSA, and the factors that affect the adoption of this behavior.

## 2. Materials and Methods

### 2.1. Study Type

This is a cross-sectional study among students of Jazan University during the period March–August 2021.

### 2.2. Study Population

Jazan University is based in the city of Jazan of the Jazan region, which is located at the southern border of the Kingdom of Saudi Arabia. Jazan University has many affiliated colleges distributed around the region. Founded in 2006, it is the province’s only university and one of the largest public, nonprofit institutions of higher education in the Kingdom of Saudi Arabia. The university includes 23 colleges granting bachelor’s degrees, and approximately 60,000 students are currently enrolled.

## 3. Sampling Procedure

The sample size of 1174 individuals was determined using the sample size statistical formula for cross-sectional surveys: initial sample size *n* = [(z^2^ * *p* * q)]/d^2^. Based on *p* = proportion of students who are interested in being vaccinated = 50% (as no information available about other related indicator that can be used for sample size calculation), Z = 1.96 equivalent to 95% confidence interval, d = error not more than 3%, and a 10% non-response rate. 

For sample size implementation, we divided the colleges of Jazan University into two main groups, a health-related group (e.g., medicine and public health) and a non-health-related group (e.g., computer sciences and arts). From each group, two colleges were chosen randomly, i.e., the Faculty of Medicine and Faculty of Applied Medical Sciences for the health-related group and Business Administration and Engineering for the non-health-related group. A web-based survey using an electronic Google Form questionnaire was used for data collection. The questionnaire link was distributed via an online social platform (WhatsApp) to the undergraduate students in the selected Jazan university college. The students’ class leaders invited students in the different selected colleges to participate in this survey.

## 4. The Study Tool

The questionnaire was based on the Health Belief Model [24]. According to this model, in order for an individual to engage in an action to avoid a certain disease, he or she should believe that (1) they are personally susceptible to the condition (the construct of perceived susceptibility), (2) that the disease is severe enough to cause concern (perceived severity), (3) taking a particular action is of benefit in reducing susceptibility or severity (perceived benefit), (4) that it would not entail overcoming potential barriers (perceived barriers), as well as (5) the confidence in the ability to perform a task or achieve a goal (self-efficacy), and (6) whether there are triggers to acting on the behavior (cues to action) [24]. The questionnaire also included some background characteristics of the participants. HBM has been extensively reported in the literature since its early adoption and has shown good results in predicting the uptake of health services [25,26].

The questionnaire was sent to four experts with interest in behavioral research for comments. Their comments resulted in some adjustments in the constructs. The questionnaire was then pilot tested among students from colleges from both strata who were not included in the study. Study Questionnaire Link: https://docs.google.com/forms/d/e/1FAIpQLScjxz0cvBdHTb2P0vzWIVjvt1tZ8omR5lqbMoydjhZ7hm4mHQ/viewform (accessed on 18 January 2022). Some minor changes were made to the wording of some questions. Reliability based on Cronbach’s alpha was calculated for the six domains of the HBM and ranged from 0.48 to 0.80 for the final version of the questionnaire. 

Ethical issues were addressed in accordance with the Helsinki Declaration and Saudi Bioethics standards’ guidelines. Approval was obtained from the Standing Committee for Scientific Research Ethics-Jazan University (HAPO-10-Z-001) reference (REC42/1/072). Consent was obtained as a prerequisite for continuation at the start of the online questionnaire. Access to the data was restricted to the research team.

## 5. Statistical Analysis 

The software Statistical Package for Social Sciences (SPSS) version 25 was used for data analysis. The electronic data were exported to the SPSS computer software. Descriptive statistics based on frequencies, percentages, averages, and standard deviations were used to describe the study population characteristics. Relationships between the dependent variable “Desire to be vaccinated” (yes/no), and a set of independent variables were examined by univariate analyses, using either t-tests on independent samples or Chi squared tests. Logistic regression analysis was used to determine factors influencing acceptance of the COVID-19 vaccine. Odds ratios and their 95% confidence intervals were calculated for each separate variable. A *p* value < 0.05 was regarded as statistically significant.

## 6. Results

The response rate for this survey was 88.5% (1039 out of 1174). Table 1 shows some background characteristics of the study participants. More of the respondents were females (approximately 60%), and three-quarters of respondents were from the 21–25-year-old age group. Respondents from the 4th academic year and medical school exhibited the highest representations in their relevant categories (each constituting approximately 40%), and the overwhelming majority of respondents were single. The vast majority of respondents are in favor of being vaccinated. Greater than one-third of respondents were already vaccinated, and only 16% do not intend to be vaccinated after approximately 6 weeks of vaccination availability.

Table 1 also shows the intention to get vaccinated against COVID-19 based on some selected characteristics. No significant differences are noted among the categories (gender, marital status, college, and residence), except for the 21–25-year-old age group, which reports a significantly higher intention.

Table 2 shows the individual questions of the constructs of HBM, the proportion of those who replied positively to the relevant statement among all participants and the results according to intention to get vaccinated. Bearing in mind that some questions were phrased negatively, the response with the highest agreement proportion among participants was “COVID-19 is a disease that spreads rapidly, so everybody, including me, is susceptible”, corresponding to approximately 86.5% of all participants. The lowest was “I have some experiences with vaccines for other diseases that make me decline the COVID-19 vaccine” in 15.5% of all participants. Numerous instances of significant differences according to the vaccine acceptance status are noted in the response to statements (19 out of 22 statements).

Table 3 shows the participants’ responses to the statements of the constructs of the HBM. A larger proportion of males responded positively compared with females. The difference was significant in most of the responses. However, some of the statements that reflect being in favor of getting vaccinated are phrased negatively.

The logistic regression results presented in Table 3 reveal no significant gender differences. The constructs of the health belief model are shown to be good predictors of the intention to be vaccinated. The construct “perceived severity” significantly predicts the acceptance of vaccination with an OR = 2.06 (95% CI: 1.55–2.75, *p* < 0.001). The other constructs might also predict behavior with those positively perceiving the vaccine to be of benefit being 1.88-fold more likely to be vaccinated (95% CI: 1.47–2.41, *p* < 0.001), and cues to action with an OR =1.39 (95% CI: 1.01–1.92, *p* < 0.05) might also predict behavior. Although “perceived barrier” significantly reduced the likelihood of being vaccinated by 39% (OR = 0.61, 95% CI: 0.45–0.63, *p* < 0.05), “perceived susceptibility” did not significantly predict behavior.

## 7. Discussion

This study explored the factors that can predict attitudes and practices toward vaccination for COVID-19 in the Kingdom of Saudi Arabia. The study used the health belief model, which is useful for studying the uptake of medical services and interventions. The level of acceptance of the COVID-19 vaccine was found to be relatively high, and all the constructs of the health belief model were shown to be good predictors of behavior with the exception of perceived susceptibility, which does not significantly predict behavior.

The level of vaccine acceptance among the students of Jazan University is relatively high. Specifically, 83.6% of respondents reported the desire to obtain the vaccine, including 35.5% who had already been vaccinated at the time of data collection. This finding is consistent with some of the levels cited in the literature and closely resembles levels among students surveyed in Italy [17] and China [18] with rates of greater than 80%. Compared to regional findings, the value obtained in this study was much higher than that noted for adults surveyed in some Arab countries (mainly from Jordan and Kuwait) with only a 29.4% acceptance rate [14] and higher than the national average in Saudi Arabia of 64.7% [15]. The results bear some similarity with Jordanian students who are undecided (25.5%) [16] and those who will “decide later” in our study (24.1%). However, the intention to get vaccinated is much higher among our study participants (83.3%) compared with respondents reporting “yes” (34.9%) and “maybe” (25.5%) in the same study (70.4% in total).

Regarding gender, the multivariate logistic regression analysis did not suggest significant differences in the tendency to be vaccinated between males and females. In contrast, most literature reported that more males are in favor of vaccination, which is consistent with that noted in the recent review of [27] and a study in Jordan and Kuwait [14].

The 21–25-year-old age groups reported the highest intentions to be vaccinated, and the logistic regression analysis further supported that they had high odds ratios. This finding is consistent with published literature where older age groups were found to be more accepting of vaccination.

The HBM constructs were good predictors of vaccine acceptance behavior with the exception of the construct “perceived susceptibility”, which did not significantly predict vaccine acceptance. Self-efficacy reflects an individual’s perception of his or her ability to perform a behavior successfully. All statements regarding this construct had a statistically significant association with the vaccine acceptance behavior (all are with *p* values <0.001). This might be a result of the well-organized access to the service and information in the study area. Cues to actions are what trigger the individual to engage in a behavior. an internal cue of having some experiences with vaccines for other diseases and external cues (with the exception of having an acquaintance who suffered from COVID-19) are all shown to be good predictors of the behavior (all have *p* values <0.001). Other studies showed that the HBM constructs predict behavior with the exception of “perceived severity” [25,26].

## 8. Limitations

Our study has some limitations. First, the study was based on cross-sectional studies, which might limit the interpretation of any association revealed by this study. Associations from the cross-sectional investigation may be regarded as less definitive compared with other study types. Second, the students’ responses may be affected by recall bias. Finally, the convenience sampling employed in the study and the unequal response rate in the different selected colleges might limit the generalizability of the study results. Despite these limitations, our research still depicts COVID-19 vaccine acceptance among university students.

## 9. Conclusions and Recommendations

In conclusion, vaccination uptake among the study population, including those who have already been vaccinated and those intending to be vaccinated, is relatively high. The Health Belief Model constructs can serve as good predictors of vaccine acceptance with the exception of “perceived susceptibility”.

The constructs and individual statements that had significant differences between those with positive and negative behaviors and attitudes can shape future interventions to enhance vaccination uptake. For example, the accumulated information on the effect of vaccination on morbidity and severity can be used to promote the uptake of vaccination based on the significant differences between the two groups in two items of the perceived benefit construct. As vaccine acceptance can vary over time, further studies of a larger population in the near future might yield useful additions to our current understanding of vaccine acceptance as well as evaluations of interventions based on the obtained information.

## Figures and Tables

**Table 1:** Intention to get vaccinated against COVID-19 according to some selected characteristics (*n* = 1039).

Characteristic		Total N%	Intended to Get Vaccinated N%		*p* Value ^1^
			Yes 803(83.6)	No 158(16.4)	
Gender	Male	419(40.3)	311(85.4)	53(14.6)	0.219
	Female	620(59.7)	492(82.4)	105(17.6)	
Age Groups	15–20	174(16.7)	131(76.6)	40(23.4)	0.008
	21–25	780(75.1)	612(85.7)	102(14.3)	
	26–30	85(8.2)	60(78.9)	16(21.1)	
Study Level	1st year	84(8.1)	63(75.0)	21(25.0)	0.385
	2nd year	117(11.3)	96(85.7)	16(14.3)	
	3rd year	164(15.8)	134(86.5)	21(13.5)	
	4th year	413(39.7)	319(83.7)	62(16.3)	
	5th year	112(10.8)	69(81.2)	16(18.8)	
	6th year	52(5.0)	42(85.7)	7(14.3)	
	Internship	97(9.3)	80(84.2)	15(15.8)	
Mode of Living	Urban	187(41.8)	359(83.7)	70(16.3)	0.926
	Rural	251(56.2)	444(83.5)	88(16.5)	
Marital Status	Married	161(15.5)	121(82.3)	26(17.7)	0.895
	Single	861(82.9)	668(83.8)	129(16.2)	
	Divorced/Widowed	17(1.6)	14(82.4)	3(17.6)	
Colleges	Medicine	419(40.3)	340(84.8)	61(15.2)	0.277
	Applied Medical Sciences	293(28.2)	222(83.8)	43(16.2)	
	Business Administration	176(16.9)	128(78.5)	35(21.5)	
	Engineering	151(14.5)	113(85.6)	19(14.4)	
HBM	Domains		Mean (SD)	Mean (SD)	*p* value ^2^
	Perc Susceptibility		2.4(0.9)	2.9(0.9)	<0.001
	Perc severity		2.6(0.7)	2.8(0.8)	0.010
	Perc Benefit		2.4(0.7)	2.8(0.7)	<0.001
	Perc Barrier		2.6(0.6)	3.5(0.7)	<0.001
	Self-Efficacy		1.9(0.7)	2.9(0.7)	<0.001
	Cues to action		2.4(0.6)	2.9(0.9)	<0.001

*p* value ^1^ based on Chi squared test. *p* value ^2^ based on independent *t* test. SD: standard deviation; HBM; health belief model.

**Table 2:** Study participant responses on items of the health belief model according to the intention to be vaccinated.

Statements		Proportion with Agree Responses N%			
		All Students	Intended to Get Vaccinated		*p* Value *
			Yes	No	
Perc Susceptibility	I do not go out much, so there is little chance that I get infected with COVID-19	371(38.6)	290(36.1)	81(51.3)	<0.001
	Because of the low spread of COVID-19 in my community, it is improbable that I get COVID-19	225(23.4)	173(21.5)	52(32.9)	0.002
	I do not mingle with those infected with COVID-19, hence, I am not going to catch the disease	288(30.0)	222(27.6)	66(41.8)	<0.001
	COVID-19 is a disease that spreads rapidly, so everybody, including me are susceptible	831(86.5)	715(89.0)	116(73.4)	<0.001
Perc Severity	COVID-19 manifestations are more severe in some age groups, to which I do not belong	390(40.6)	324(40.3)	66(41.8)	0.739
	COVID-19 causes a severe illness that is more dangerous than Influenza	768(79.9)	655(81.6)	113(71.5)	0.004
	I follow some healthy habits that will make the disease less severe in case I get infected	368(38.3)	299(37.2)	69(43.7)	0.123
Perc Benefit	Vaccination against COVID-19 will decrease the chances of infection if I had a contact with infected persons	660(68.7)	609(75.8)	51(32.3)	<0.001
	Vaccination against COVID-19 will decrease the severity of the illness if I get infected	701(72.9)	644(80.2)	57(36.1)	<0.001
	I think that there are some other ways, which are more effective than vaccination	310(32.3)	220(27.4)	90(57.0)	<0.001
Perc Barrier	COVID-19 vaccination might cause severe side effects that might cause great damage to my health	406(42.2)	298(37.1)	108(68.4)	0.001
	Some vaccination side effects, which are not apparent, might appear sometime later in the future	260(27.1)	182(22.7)	78(49.4)	<0.001
	I will be able to get the vaccine, even if it is not available for free	471(49.0)	432(53.8)	39(24.7)	<0.001
	I do not mind if vaccination is done only by injections or the like, even if there is some pain	667(69.4)	620(77.2)	47(29.7)	<0.001
Self-Efficacy	I will be able to go to the health center more than once to get as many shots as necessary for full immunity	707(73.6)	644(80.2)	63(39.9)	<0.001
	I will be able to reach the health center to get vaccinated	793(82.5)	713(88.8)	80(50.6)	<0.001
	I have the ability to bear the possible side effects of COVID-19	620(64.5)	579(72.1)	41(25.9)	<0.001
	I have the ability to obtain information about COVID-19 vaccine and updates	715(74.4)	632(78.7)	83(52.5)	<0.001
Cues to Action	I have those who died or suffered from COVID-19 among my relatives or acquaintances	475(49.4)	400(49.8)	75(47.5)	0.590
	I have sufficient information on how vaccines work to protect against diseases	686(71.4)	611(76.1)	75(47.5)	<0.001
	A lot of my relatives and acquaintances had already been vaccinated	654(68.1)	582(72.5)	72(45.6)	<0.001
	I have some experiences with vaccines for other diseases that makes me decline the COVID-19 vaccine	153(15.9)	109(13.6)	44(27.8)	<0.001

* p value based on Chi squared test.

**Table 3:** Multivariate logistic regression analysis of independent predictors of the acceptance of COVID-19 vaccination.

Variables	*p* Value	$O\hat{R}$	$95\%\;C.I.\;\mathrm{for}\;(O\hat{R})$	
			Lower	Upper
Gender				
Female (ref)				
Male	0.204	1.29	0.75	1.96
Age groups (years)				
15–20 (ref)				
21–25	<0.001	2.54	5.69	2.54
26–30	0.023	1.12	4.48	1.12
HBM Covariates				
Perc Susceptibility	0.564	0.93	0.74	1.18
Perc Severity	<0.001	2.06	1.55	2.75
Perc Benefit	<0.001	1.88	1.47	2.41
Perc Barrier	0.002	0.61	0.45	0.83
Self-Efficacy	<0.001	0.36	0.27	0.49
Cues to Action	0.042	1.39	1.01	1.92

$O\hat{R}$, estimated ODDs ratio, 95 % C.I. = 95% confidence interval.

## Data Availability

This study has no additional supporting data to share.
